# The novel Nsp9-interacting host factor H2BE promotes PEDV replication by inhibiting endoplasmic reticulum stress-mediated apoptosis

**DOI:** 10.1186/s13567-023-01158-w

**Published:** 2023-03-22

**Authors:** Xingang Xu, Mingrui Ma, Xiaojie Shi, Yuchao Yan, Yi Liu, Naling Yang, Quanqiong Wang, Shuxia Zhang, Qi Zhang

**Affiliations:** College of Veterinary Medicine, Northwest A&F University, Yangling, 712100 Shaanxi China

**Keywords:** PEDV, HIST2H2BE, Nsp9, ER stress, apoptosis, virus replication

## Abstract

Porcine epidemic diarrhoea (PED) caused by porcine epidemic diarrhoea virus (PEDV) has led to significant economic losses in the swine industry worldwide. Histone Cluster 2, H2BE (HIST2H2BE), the main protein component in chromatin, has been proposed to play a key role in apoptosis. However, the relationship between H2BE and PEDV remains unclear. In this study, H2BE was shown to bind and interact with PEDV nonstructural protein 9 (Nsp9) via immunoprecipitation-mass spectrometry (IP-MS). Next, we verified the interaction of Nsp9 with H2BE by immunoprecipitation and immunofluorescence. H2BE colocalized with Nsp9 in the cytoplasm and nuclei. PEDV Nsp9 upregulated the expression of H2BE by inhibiting the expression of IRX1. We demonstrated that overexpression of H2BE significantly promoted PEDV replication, whereas knockdown of H2BE by small interfering RNA (siRNA) inhibited PEDV replication. Overexpression of H2BE led to significantly inhibited GRP78 expression, phosphorylated PERK (p-PERK), phosphorylated eIF2 (p-eIF2), phosphorylated IRE1 (p-IRE1), and phosphorylated JNK (p-JNK); negatively regulated CHOP and Bax expression and caspase-9 and caspase-3 cleavage; and promoted Bcl-2 production. Knocking down H2BE exerted the opposite effects. Furthermore, we found that after deletion of amino acids 1–28, H2BE did not promote PEDV replication. In conclusion, these studies revealed the mechanism by which H2BE is associated with ER stress-mediated apoptosis to regulate PEDV replication. Nsp9 upregulates H2BE. H2BE plays a role in inhibiting apoptosis and thus facilitating viral replication, which depends on the N-terminal region of H2BE (amino acids 1–28). These findings provide a reference for host–PEDV interactions and offer the possibility for developing strategies for PEDV decontamination and prevention.

**Supplementary Information:**

The online version contains supplementary material available at 10.1186/s13567-023-01158-w.

## Introduction

Porcine epidemic diarrhoea (PED) is an acute contact-mediated infectious intestinal disease of pigs caused by porcine epidemic diarrhoea virus (PEDV) [1]. The clinical symptoms of the disease are mainly diarrhoea, vomiting and dehydration, cause high mortality in piglets and has led to large economic losses to the swine industry [2]. PEDV is a single-stranded positive-sense RNA virus that belongs to the *Alphacoronavirus* genus in the Coronaviridae family [3]. The whole-genome length of PEDV is 28 kb, which includes a 5′ untranslated region (5′ UTR), a 3′ UTR and a poly (A) tail, and seven open reading frames (ORFs) encoding four major structural proteins (S, E, M, N proteins) and the auxiliary protein ORF3. ORF1a and ORF1b encode two multiprotein precursors that, upon cleavage by proteases, produce a series of nonstructural proteins (from Nsp1 to Nsp16). Research has suggested that ORF3 facilitates PEDV proliferation through a mechanism that inhibits the apoptosis of infected cells [4]. PEDV Nsp2 can hamper host innate antiviral response activation and promote PEDV replication by targeting FBXW7 for degradation [5]. However, the interaction of PEDV Nsp9 with host proteins is not known.

The endoplasmic reticulum (ER) is the site of protein posttranslational modification and cellular protein folding. Many cellular stress conditions, such as hypoxia, a lack of glucose and viral infection, lead to unfolded or misfolded protein accumulation in the ER lumen, which can result in ER stress [6]. In response to ER stress, cells activate a self-defence mechanism, the unfolded-protein response (UPR) [7]. Misfolded proteins are eventually retained in the ER lumen and eliminated by the ER-related degradation (ERAD) pathway or with the death of the cell via apoptosis [8].

Apoptosis is the programmed death of cells regulated mainly by caspase family proteins, maintaining intracellular homeostasis by removing damaged and infected cells [9]. Apoptosis is activated through three different pathways: the extrinsic pathway, the intrinsic pathway, and the ER stress pathway. One of the main mechanisms of ER stress-mediated apoptosis is the induction of CHOP expression [10]. ZIKV inhibits apoptosis by suppressing the expression of CHOP/DDIT3 [11]. Our previous studies have shown that CCN1 inhibits viral replication by promoting PEDV-induced apoptosis via the mitochondrial pathway [12]. However, the mechanism by which apoptosis affects PEDV replication is not completely clear.

Histone Cluster 2, H2BE (HIST2H2BE) is a variant of histone H2B (referred to herein as H2BE). Histones are involved in many cellular processes, such as DNA replication, repair, recombination, transcriptional regulation and apoptosis. H2BE can interact with the DNASE1L3 protein in the nucleus and clear the accumulated damaged DNA from the cytoplasm [13]. In addition, upregulation of H2BE in tumour cells inhibit the apoptosis of cancer cells and promote tumour formation [14]. A study showed that H2B is an important component of genome recognition post KSHV or HSV-1 infection to induce IFN-β production [15]. However, the role of H2BE in PEDV replication is unclear.

In this study via immunoprecipitation-mass spectrometry (IP-MS), co-IP and immunofluorescence, we confirmed that H2BE interacts with PEDV Nsp9. PEDV Nsp9 promotes the expression of H2BE by inhibiting IRX1 expression. We found that H2BE positively regulates viral infection. Overexpression of H2BE promotes the replication of PEDV and inhibits ER stress-mediated apoptosis, whereas knockdown of H2BE inhibits PEDV replication and promotes ER stress-mediated apoptosis. In addition, the presence of amino acids 1–28 in the N-terminal region of H2BE is important for its functions that promote viral replication. These findings provide new insights into the regulation of PEDV replication by the host factor H2BE and may be used to develop a therapeutic strategy to control PEDV infection.

## Materials and methods

### Cell culturess

Monkey embryonic kidney epithelial cells (Marc-145 cells) were stored in our laboratory. Marc-145 cells were cultured in Dulbecco’s modified Eagle’s minimum (DMEM; HyClone, Logan, UT, USA) supplemented with 10% foetal bovine serum (FBS; Gibco, Grand Island, NY, USA). Marc-145 cells were grown in culture with 5% CO_2_ at 37 °C.

### Virus, virus titration and virus infections

The PEDV Shaanxi strain CH/SXYL/2016 was isolated and stored in our laboratory. In Marc-145 cells, the PEDV strain was amplified and titrated. In brief, 96-well plates were coated with a monolayer of Marc-145 cells before being infected with serially diluted PEDV (10^–1^ to 10^–10^). The Reed-Muench technique was used to determine the viral titre, which was then converted into a 50% tissue culture infective dose (TCID_50_). The PEDV strain was used to infect Marc-145 cells at a multiplicity of infection (MOI) of 1.

### Viral adsorption and entry

Marc-145 cells were infected with PEDV at an MOI of 1 and were placed at 4 °C for 1 h. The cells were washed three times with PBS to remove unabsorbed virus and maintained in DMEM with 2% FBS at 37 ℃ for 1 h.

### Plasmid construction and transfection

The recombinant plasmid Nsp9-EGFP was constructed by inserting the full-length sequence of PEDV Nsp9 (324 bp) into a pEGFP-C1 vector.

For HIST2H2BE overexpression, the full-length sequence of the HIST2H2BE gene (GenBank No. XM_008019209.2) was amplified from the cDNA of Marc-145 cells obtained from reverse transcription-PCR using the primer pair H2BE-F and H2BE-R and then subcloned into a pcDNA3.1-Flag vector to generate H2BE-Flag. The H2BE∆N-Flag, H2BE∆C-Flag and H2BE-Δ1-28-Flag recombinant plasmids were constructed by inserting the H2BE-∆N (1 to 192 bp), H2BE-∆C (193 to 378 bp) and H2BEΔ1-28 (1 to 84 bp) deletion mutant sequences into a pcDNA3.1-Flag vector. The full-length sequence of the IRXI gene (GenBank No. XM_007961160.2) was cloned into a pcDNA3.1-Flag vector. The plasmids were sequenced by Augct Company.

Eukaryotic expression plasmids were transfected into Marc-145 cells using Lipo8000™ transfection reagent (Beyotime Biotechnology, Shanghai, China) according to the manufacturer’s instructions. The efficiency of protein overexpression was determined by Western blotting. All PCR primers are listed in Table 1.

**Table 1:** **List of primers for PCR**

Primer name	Sequence (5′-3′)
H2BE-F	CGGGATCCGCCACCATGCCTGAACCGGCAAAATC
H2BE-R	GCCTCTAGACTTGGAGCTGGTGTACTTGGT
H2BE-∆N F	CGGGATCCGCCACCATGCCTGAACCGGCAAAATC
H2BE-∆N R	CGTCTAGAGTTCATGATGCCCATGGCCTT
H2BE-∆C F	GGAATTCGCCACCATGTCCTTCGTCAACGACATT
H2BE-∆C R	CGGATCCTGCTTGGAGCTGGTGTACTTGGT
H2BE-∆1–28 F	CGGGATCCGCCACCATGGAGAGCTACTCCATCTACG
H2BE-∆1–28 R	GCTCTAGACTTGGAGCTGGTGTACTTGGTG
Nsp9-F	GCAAGCTTGCCACCATGAATAATGAAATTATTCCTGG
Nsp9-R	CGGATCCCTGCAAGCGTACAGTGGC
IRX1-F	GCGAATTCGCCACCATGTCCTTCCCGCAGCTGGGCTAC
IRX1-R	GCTCTAGAAAGGCGGACGGGAGGGCTGCTAGGAT

### Small interfering RNA knockdown and transfections

We designed and synthesized three small interfering RNAs (siRNAs) and one negative control (siNC) targeting the H2BE protein at the General Biol Company (Anhui, China). Following the manufacturer’s guidelines, Marc-145 cells were cultivated to 60–70% confluence in 6-well cell culture plates, and siRNA or siNC was transfected using Lipo8000™ (Beyotime Biotechnology, Shanghai, China). At 36 h post-transfection, the cells were infected with PEDV and then collected at different time points. Western blotting was performed to evaluate the efficiency of gene knockdown. The sequences of siH2BE and siNC are listed in Table 2.

**Table 2:** **Sequences of siRNAs used in this study**

Primer name	Sequence (5′-3′)
siH2BE-1F	CCAAGGCGGUCACCAAGUACATT
siH2BE-1R	UGUACUUGGUGACCGCCUUGGTT
siH2BE-2F	AGGAGAGCUACUCCAUCUACGTT
siH2BE-2R	CGUAGAUGGAGUAGCUCUCCUTT
siH2BE-3F	CCAAGAAAGCCGUCACCAAAGTT
siH2BE-3R	CUUUGGUGACGGCUUUCUUGGTT
NC-si–F	UUCUCCGAACGUGUCACGUdTdT
NC-si-R	ACGUGACACGUUCGGAGAAdTdT
siIRX1-F	GCAUCGACAUUGACAAGAUTT
siIRX1-R	AUCUUGUCAAUGUCGAUGCTT

### Inhibitors and antibodies

The ER stress inhibitor 4-phenylbutyrate (4-PBA) and a pancaspase inhibitor were obtained from MCE (Shanghai, China). All inhibitors were solubilized in dimethyl sulfoxide (DMSO; Solarbio, Beijing, China). Inhibitors were diluted, added to culture medium, and cultivated for an hour before infection. Antibodies against Bax, Bcl-2, caspase-3, caspase-9 and CHOP were purchased from Cell Signaling Technology (Danvers, MA, USA). Primary antibodies against GRP78, PERK, p-PERK, eIF2, p-eIF2, IRE1, p-IRE1, JNK, p-JNK and β-actin were purchased from ABclonal (Wuhan, China). An antibody against IRX1 was purchased from ABmart (Shanghai, China), and antibodies against Flag-tagged, GFP-tagged and PE-conjugated anti-mouse IgG were purchased from TRAN (Beijing, China). A polyclonal antibody directed against the PEDV (CH/SXYL/2016) N protein and PEDV Nsp9 protein was prepared in our laboratory.

### RNA isolation and quantitative real-time PCR (qRT‒PCR) analysis

TRIzol Universal (TIANGEN, China) was used to extract total RNA from Marc-145 cells, which was subsequently reverse-transcribed into cDNA using a FastKing RT Kit (TIANGEN, China) following the manufacturer’s guidelines. SYBR Green Select Master Mix (ABclonal, China) was used for qPCR analysis on a Real-Time PCR Detection System (TIAN LONG, China). Relative expression levels of target genes were adjusted using β-actin as the internal standard and the 2^−ΔΔCT^ method. All qPCR primers are listed in Table 3.

**Table 3:** **List of primers for qPCR**

Primer name	Sequence (5′-3′)
H2BE-qF	TCCAAGAAAGCCGTCACCAA
H2BE-qR	GACCTGCTTCAGCACCTTGT
PEDV-qF	AGATCGCCAGTTTAGCACCA
PEDV-qR	GGCAAACCCACATCATCGT
IFN-β-qF	ACGGCTCTTTCCATGAGCTAC
IFN-β-qR	GTCAATGCAGCGTCCTCCTT
ISG15-qF	CACCGTGTTCATGAATCTGC
ISG15-qR	CTTTATTTCCGGCCCTTGAT
TET1-qF	GATGACAGAGGTTCTTGCACAT
TET1-qR	AGGTTGCACGGTCTCAGTGT
IRX1-qF	GGAATGTGGGAGGAATTAAGAC
IRX1-qR	GCATTTACCGAACCCGATA
LC3-qF	CATCCAACCAAAATCCCG
LC3-qR	GTGGCCGTTCACCAACAG
β-Actin-qF	CTTAGTTGCGTTACACCCTTTC
β-Actin-qR	TGTCACCTTCACCGTTCCA

### Western blot analysis

The cells were lysed in radioimmunoprecipitation assay (RIPA) buffer with 1% PMSF, and then the protein concentration was quantified using a BCA protein assay reagent kit (Pierce, Rockford, USA). The lysed products were heated at 95 ℃ and maintained at this temperature for 10 min. Then, the same amount of protein samples was resolved by 10–15% SDS‒PAGE. The proteins were then transferred to a polyvinylidene fluoride (PVDF) membrane (Roche, Basel, Switzerland). Next, the membrane was blocked with TBST buffer supplemented with 5% skim milk (China) or 5% BSA (Yuanyai Biotechnology, China) for 2 h at room temperature and incubated with the corresponding primary antibody by gentle shaking overnight at 4 ℃. The protein bands were visualized using an ECL kit (GE Healthcare), and images were obtained by ImageJ software after probing with specific secondary antibodies, namely, goat anti-mouse IgG or goat anti-rabbit IgG.

### Apoptotic rate measurement

The effects of siH2BE on cell apoptosis were detected using an Annexin V-FITC apoptosis kit (BioVision, Inc., CA, USA) according to the manufacturer’s protocol. Briefly, the cells were treated with trypsin, collected, and washed twice with prechilled PBS, and then, the cells were counted. Next, the cells were resuspended in 500 μL of Annexin V-FITC binding buffer, 5 μL of Annexin V-FITC and 10 μL of propidium iodide (PI) was added to the suspension, and the cells were incubated for 15 min at room temperature in the dark. The percentage of positive cells was determined after flow cytometry assay.

### Co-IP assay

To study the interaction of the PEDV Nsp9 protein with the H2BE protein and H2BE structural mutants, pEGFP-Nsp9 was cotransfected with H2BE-Flag or H2BE-Δ1-28-Flag into MARC-145 cells. At 48 h post-transfection, the cells were lysed for use in Co-IP. Total protein lysates were incubated at 4 ℃ with 2 mg of mouse anti-Flag antibody or the same amount of mouse IgG, which was the control. After overnight incubation, 40 µL of protein A + G agarose (TransGen, China) was added to the protein–antibody mixture, which was incubated at 4 °C for 12 h. After washing, the protein interaction was analysed with the eluted samples by Western blotting.

### Immunofluorescence assay

Cells were rinsed three times with PBS, fixed with 4% paraformaldehyde fixative solution for 15 min at room temperature and permeabilized with 0.1% Triton X-100 for 10 min. The samples were blocked with 5% bovine serum albumin for 2 h and incubated overnight with anti-Flag mouse monoclonal antibody (1:500) at 4 ℃. The cells were rinsed three times for 5 min each time with PBS and incubated with PE-AffiniPure goat anti-mouse IgG (1:500) for 1 h in the dark at room temperature. Cell nuclei were counterstained with DAPI (Biosharp, China) for 5 min and then rinsed three times with PBS. Finally, the treated cells were visually examined using a laser scanning confocal microscope (Leica TCS SP8, Germany).

### MTT assay

According to the manufacturer's instructions, Cell Proliferation Kit I (MTT) was used for the MTT test to measure the effect of the inhibitor on cell activity and proliferation. In 96-well plates, monolayers of Marc-145 cells were grown to 90% confluency. The inhibitor was applied to each well and incubated with the cells for 24 h at the appropriate concentration. Then, 20 μL of MTT-labelled reagents was added to each well, and the cells were incubated for 4 h at 37 °C. In an enzyme-linked immunosorbent assay reader, absorbance was measured at 550 nm following the addition of 150 μL of a solubilizing solution.

### Statistical analysis

Each of the tests was repeated three times. The information was statistically analysed using the GraphPad Prism 8 program, and results are reported as the mean and standard deviation (SD). Unpaired t tests were performed for comparisons between two sets of data. One-way analysis of variance (ANOVA) was performed to determine significant differences between experimental groups and control groups. Statistically significant differences are denoted at the level of *P ≤ 0.05; **P ≤ 0.01; and *** *P* ≤ 0.001.

## Results

### PEDV Nsp9 interacts with the host protein H2BE

**Figure 1:**
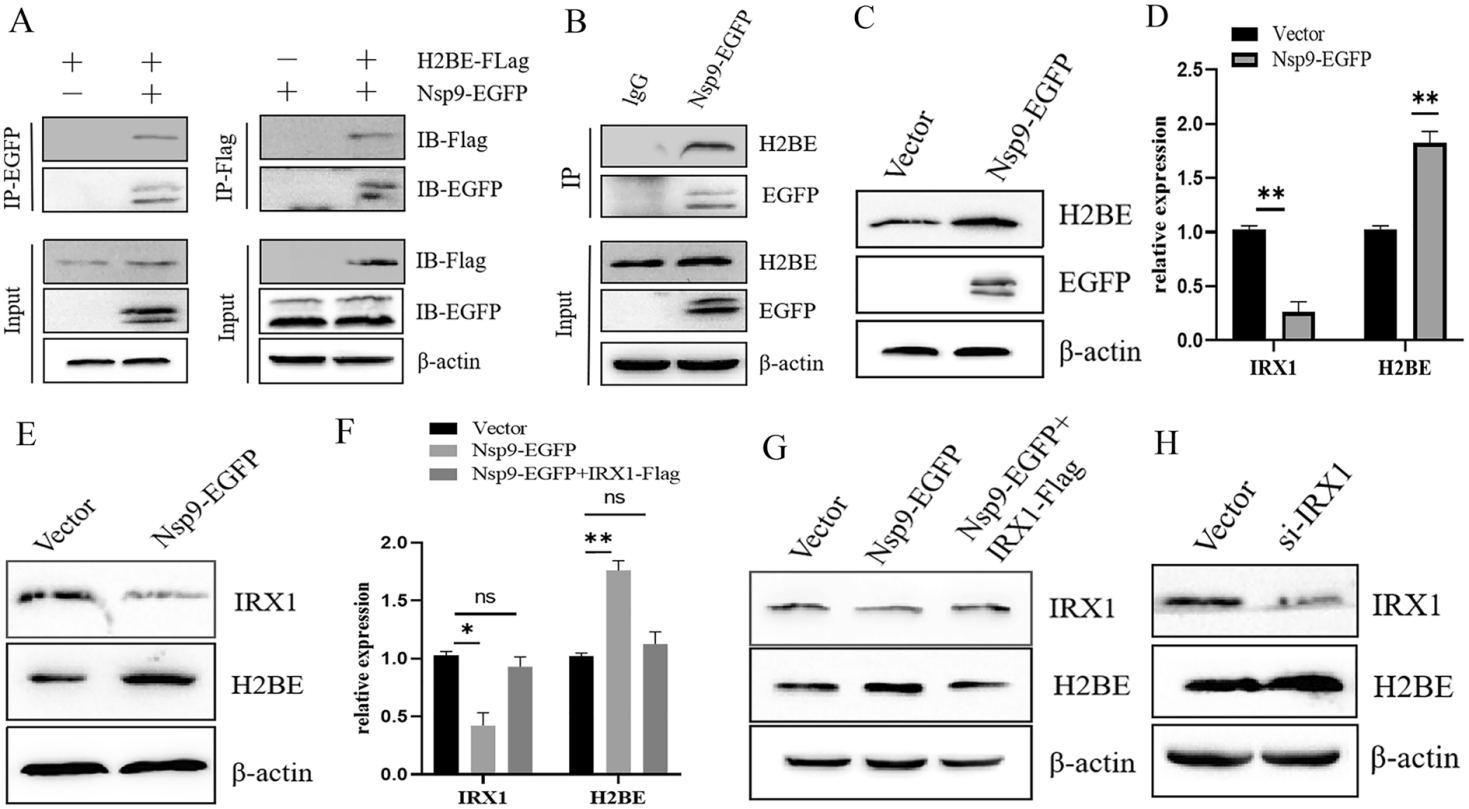
**Nsp9 interacts with H2BE.** **A** Lysates of Marc-145 cells overexpressing Nsp9-EGFP and H2BE-Flag were subjected to reciprocal coimmunoprecipitation (co-IP) performed to detect protein interactions. **B** Lysates of Marc-145 cells overexpressing Nsp9-EGFP were subjected to co-IP and immunoblotting to detect any interaction between NSP9 and endogenous H2BE. **C** Effect of PEDV NSP9 overexpression on H2BE expression as detected by Western blotting. **D** IRX1 and H2BE mRNA expression levels were measured by qPCR. β-Actin was used as the internal control. **E** Western blot analysis was performed to assess the inhibition of IRX1 expression after Nsp9 overexpression in Marc-145 cells. **F** IRX1 and H2BE mRNA expression levels were measured by qPCR. β-Actin was used as the internal control. **G** Western blotting was performed to measure the expression of IRX1 and H2BE in Marc-145 cells under the indicated conditions. Marc-145 cells were transfected with si-IRX1 or NC-si, and the cells were collected 36 h later. **H** The protein levels of IRX1 and H2BE in si-IRX1-transfected MARC-145 cells were analysed by Western blotting. The results are representative of three independent experiments. The data are presented as the mean ± SD, *n* = 3 (*P < 0.05; **P < 0.01).

The interaction mechanisms of PEDV structural proteins and various nonstructural proteins with host cell proteins to regulate PEDV replication have been extensively studied, but the interaction mechanisms of PEDV Nsp9 with host cells remain unclear. To investigate whether host factors interact with Nsp9 to regulate the replication of PEDV, Marc-145 cells were transfected with the Nsp9-EGFP plasmid for 48 h, and then, a coimmunoprecipitation assay was performed with an anti-GFP antibody. Specific migrating bands of Nsp9-coimmunoprecipitated proteins in Western blots were excised for IP-MS analysis (data not shown). The results showed that histone H2BE may bind to Nsp9. In this study, to validate the physical interactions between Nsp9 and H2BE, we performed mutual coimmunoprecipitation assays using anti-Flag and anti-GFP antibodies with Marc-145 cells transfected with Nsp9-EGFP and H2BE-Flag vectors. The results revealed that Nsp9-EGFP coimmunoprecipitated with H2BE-Flag (Figure 1A). In addition, coimmunoprecipitation using Marc-145 cell lysates verified the specific interaction of Nsp9 with endogenous H2BE (Figure 1B). Furthermore, we transfected the Nsp9-EGFP vector into Marc-145 cells and collected the cells after 36 h in culture for Western blotting analysis to explore whether Nsp9 regulates the intracellular level of H2BE. We found that the protein levels of H2BE were increased (Figure 1C). IRX1 has been reported to be involved in the regulation of H2BE expression [14]. To investigate whether Nsp9 upregulates H2BE expression by inhibiting IRX1 expression, we examined the expression of IRX1 in Marc-145 cells transfected with the Nsp9-EGFP vector. The results showed that the mRNA and protein levels of IRX1 were significantly lower in Nsp9-EGFP-transfected samples than in vector samples (Figures 1D and E), and overexpression of IRX1 downregulated H2BE levels in Nsp9-EGFP-transfected cells (Figures 1F and G). To verify that Nsp9 upregulated the expression of H2BE by inhibiting IRX1 expression, small interfering RNA of IRX1 was synthesized to knockdown IRX1 in Marc-145 cells. As shown in Figure 1H, inhibition of IRX1 upregulated the expression of H2BE. In summary, these data suggest that Nsp9-EGFP upregulates H2BE expression in Marc-145 cells by inhibiting IRX1 expression.

### Upregulation of H2BE expression in PEDV-infected cells

**Figure 2:**
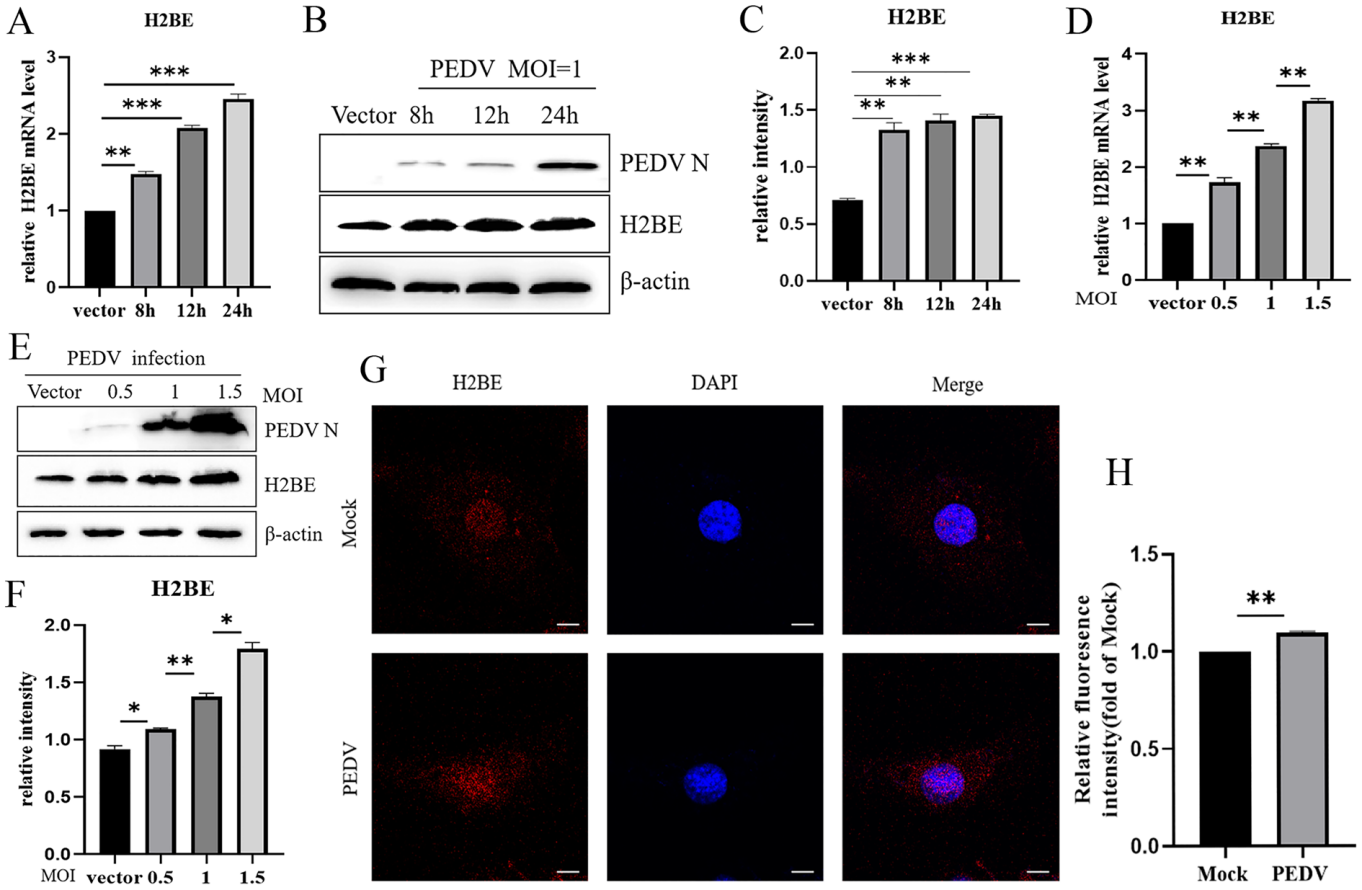
**Expression of H2BE after PEDV infection in Marc-145 cells**. Marc-145 cells were infected or mock-infected with PEDV at an MOI of 1 and harvested 8 h, 12 h and 24 h later. **A** H2BE mRNA expression levels were detected and analysed by qPCR. β-Actin was used as the internal control. **B** H2BE protein expression levels were measured by Western blotting. **C** The intensity represents the H2BE protein level normalized to that of β-actin. Marc-145 cells were infected with PEDV at the indicated MOI (0, 0.5, 1 or 1.5) and harvested 24 h later. **D** H2BE mRNA expression levels were measured by qPCR. **E** H2BE protein expression levels were measured by Western blotting. **F** The intensity represents the H2BE protein level normalized to that of β-actin. β-Actin was used as the internal control. Marc-145 cells were infected or mock-infected with PEDV at an MOI of 1 and harvested 24 h later. Cells were fixed and incubated with rabbit anti-H2BE antibody (1:500). **G** Immunofluorescence assays were performed to further analyse the intracellular expression of H2BE. **H** Analysis of H2BE protein fluorescence intensity. The results are representative of three independent experiments. The data are presented as the mean ± SD, *n* = 3, (*P < 0.05; **P < 0.01).

To explore the regulation of H2BE expression by PEDV infection, Marc-145 cells were infected with PEDV at an MOI of 1 or mock-infected with PEDV for 8 h, 12 h and 24 h, and we measure the mRNA level of H2BE by qPCR and the protein level by Western blotting. We found that the mRNA level of H2BE significantly increased after 8 h, 12 h and 24 h (Figure 2A). Similarly, the protein level was significantly increased (Figures 2B and C). To determine whether H2BE expression was increased in a PEDV dose-dependent manner, we infected Marc-145 cells with PEDV at an MOI of 0.5, 1, and 1.5 and collected samples 24 h after infection. The data indicated that the H2BE mRNA level (Figure 2D) and protein level were increased (Figures 2E and F) in a dose-dependent manner. Furthermore, Marc-145 cells were infected with PEDV at an MOI of 1 or mock-infected with PEDV for 24 h. We observed the expression of H2BE in the PEDV-infected Marc-145 cells by indirect immunofluorescence. The PEDV infection group exhibited higher levels of H2BE protein than the mock group (Figures 2G and H). Taken together, these results suggest that PEDV infection upregulates H2BE mRNA and protein expression in Marc-145 cells.

### Overexpression of H2BE promotes PEDV reproduction

**Figure 3:**
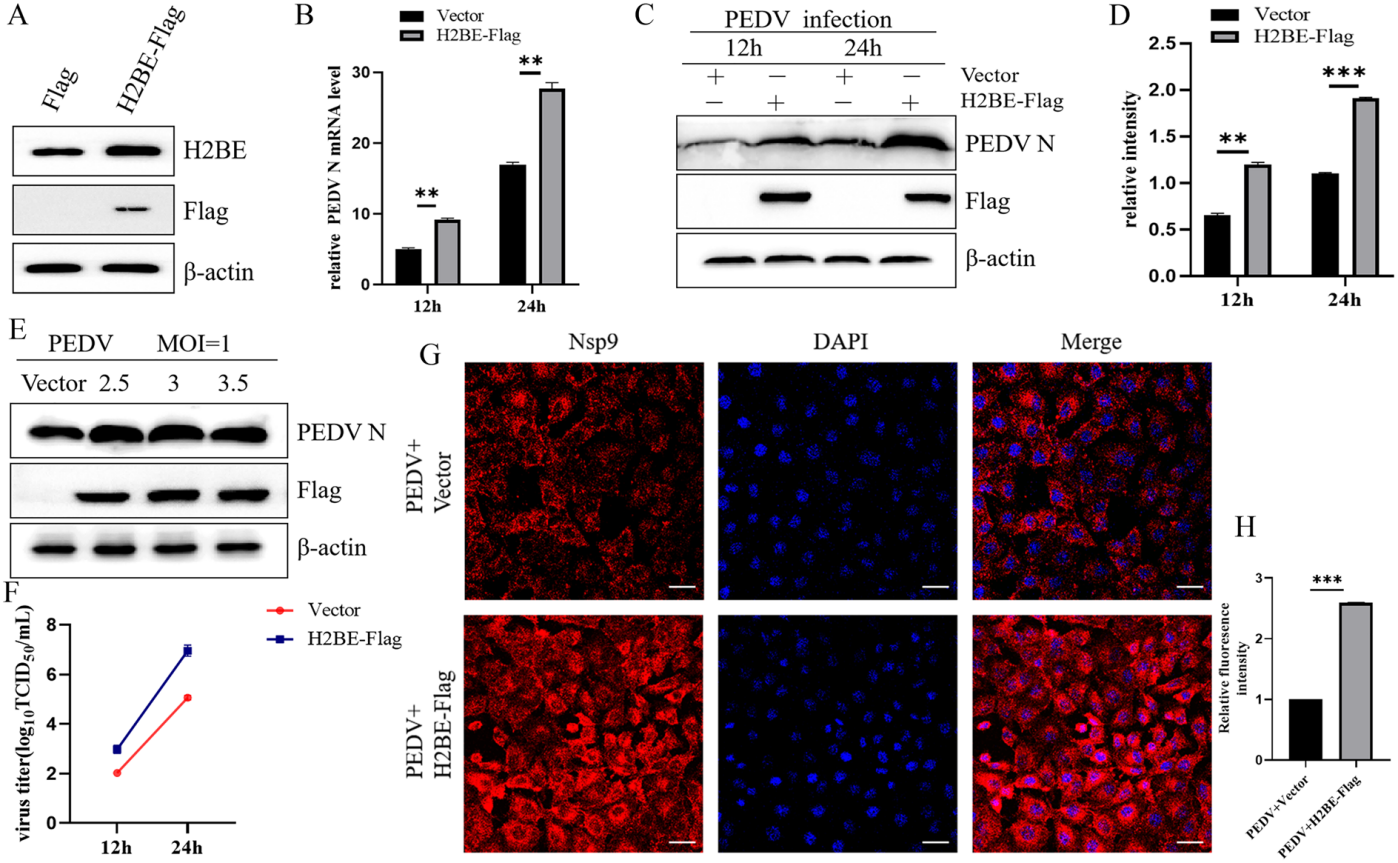
**Overexpression of H2BE facilitates PEDV replication.** Marc-145 cells were transfected with H2BE-Flag or am empty vector. **A** High-level expression of H2BE was confirmed by Western blotting. Marc-145 cells were transfected with H2BE-Flag (2.5 μg) or the empty vector (2.5 μg) and then infected with PEDV at an MOI of 1 and harvested 12 h and 24 h later. **B** The PEDV N protein level was measured by Western blotting. **C** PEDV N mRNA was detected and analysed by qPCR. β-Actin was used as the internal control. **D** The intensity represents the PEDV N protein level normalized to that of β-actin. Marc-145 cells were transfected with H2BE-Flag (0, 2.5, 3 and 3.5 μg) and then infected with PEDV at an MOI of 1 and harvested 24 h later. **E** The PEDV N protein level was measured by Western blotting. **F** PEDV titres in the culture supernatants were determined using the TCID_50_ method. The results are representative of three independent experiments. The data are presented as the mean ± SD, *n* = 3, (*P < 0.05; **P < 0.01). **G** Marc-145 cells were transfected with H2BE-Flag or the empty vector and then infected with PEDV at an MOI of 1.0. Cells were fixed and incubated with mouse anti-PEDV Nsp9 polyclonal sera (1:500) 24 h post-infection. Immunofluorescence assays were used to further observe intracellular propagation of PEDV. **H** Analysis of H2BE protein fluorescence intensity.

Previous studies have shown that H2B is involved in the replication of several viruses [15]. To examine the effects of H2BE on PEDV replication, we successfully overexpressed H2BE-Flag-tagged proteins in Marc-145 cells, and the overexpression efficiency was verified by Western blotting (Figure 3A). Marc-145 cells were transfected with H2BE-Flag or empty vector, infected with PEDV at 1 MOI and harvested at the specified time. As shown in Figures 3B–D, the expression levels of the PEDV N protein and mRNA in H2BE-Flag-overexpressing Marc-145 cells were significantly higher than those in cells transfected with the empty vectors. In addition, we examined the supernatant of cultured cells transfected with the H2BE overexpression plasmid, and the results showed an increase in viral titre compared to the level after transfection with the empty vector (Figure 3F). To confirm the propagation of PEDV in H2BE-Flag-overexpressing Marc-145 cells, we performed indirect immunofluorescence experiments. The H2BE-Flag overexpression group carried more PEDV virions than the empty vector group (Figures 3G and H). Then, we examined the effects of H2BE dose on PEDV protein expression. Marc-145 cells were transfected with H2BE at 2.5 μg, 3 μg and 3.5 μg; infected with PEDV at an MOI of 1; and cultured for 24 h. A Flag protein intensity analysis showed that different doses of the H2BE overexpression vector had been successfully transfected (Additional file 1). The results showed that the promoting effect of H2BE on PEDV replication was not related to the infection dose (Figure 3E). These results suggest that overexpression of H2BE promotes PEDV replication in Marc-145 cells.

### Knockdown of H2BE inhibits PEDV replication

**Figure 4:**
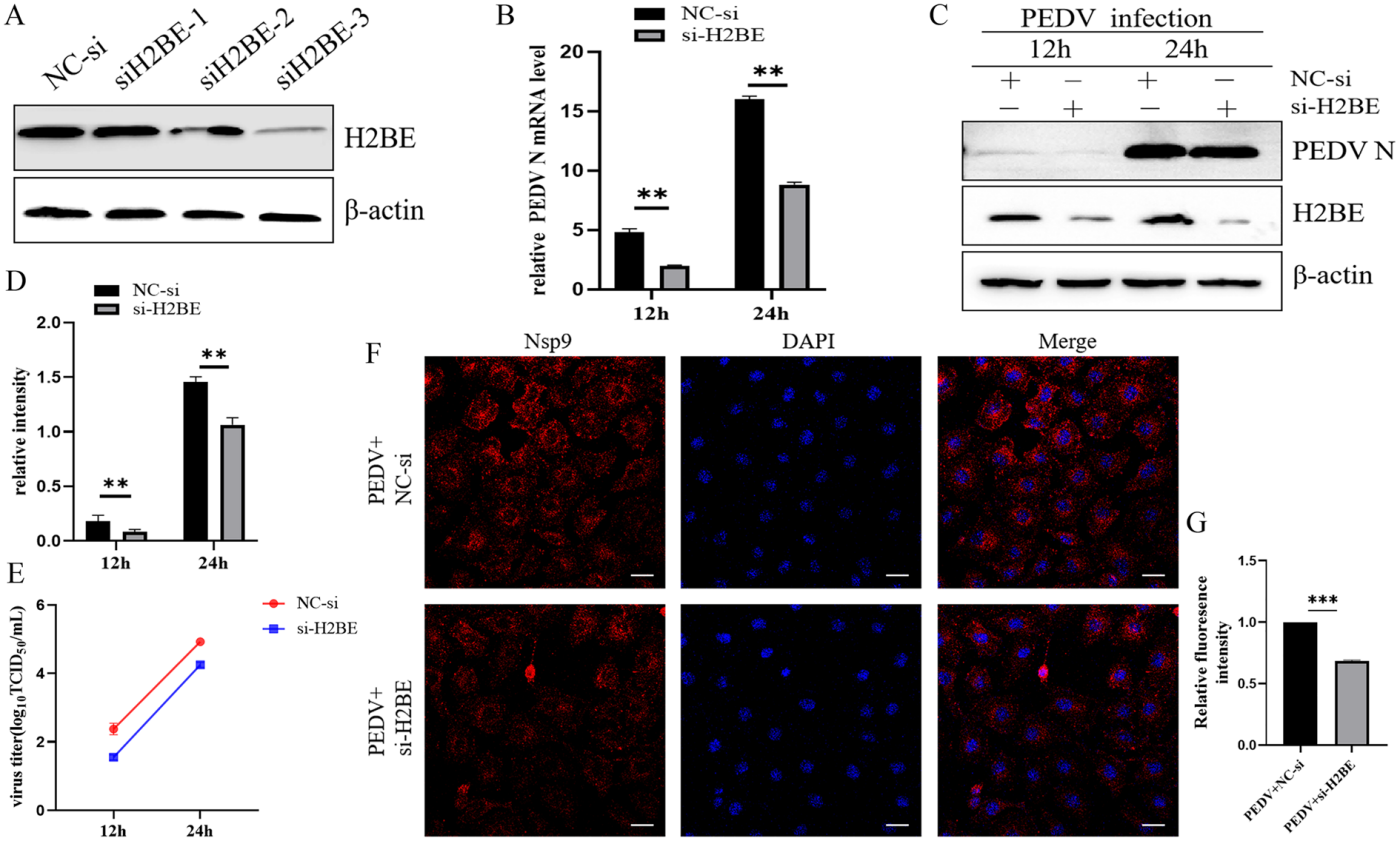
**Knockdown of H2BE inhibits PEDV replication.** **A** Three H2BE-targeting siRNAs and nontargeting control siRNA (NC-si) were transfected into Marc-145 cells, and then, the knockdown efficiencies of the three siRNAs were compared by Western blotting. Marc-145 cells were transfected with si-H2BE or NC-si and then infected with PEDV at an MOI of 1.0 and harvested 12 h and 24 h later. **B** The PEDV N protein level was measured by Western blotting. **C** PEDV N mRNA level was measured by qPCR. β-Actin was used as the internal control. **D** The intensity represents the PEDV N protein level normalized to that of β-actin. **E** PEDV titres in the culture supernatants were determined using the TCID_50_ method. The results are representative of three independent experiments. The data are presented as the mean ± SD, *n* = 3, (*P < 0.05; ** < 0.01). Marc-145 cells were transfected with si-H2BE or NC-si and then infected with PEDV at an MOI of 1.0. **F** Cells were fixed and incubated with mouse anti-PEDV Nsp9 polyclonal sera (1:500) 24 h post-infection. Immunofluorescence assays were used to further observe intracellular propagation of PEDV. **G** Analysis of H2BE protein fluorescence intensity.

To validate the functions of H2BE in PEDV infection, synthetic H2BE-specific siRNAs were transfected into Marc-145 cells, and the interference efficiency was analysed by Western blotting. As shown in Figure 4A, siH2BE-3 showed the greatest inhibitory effect on H2BE protein expression and was therefore selected for subsequent interference experiments. Knockdown of H2BE significantly inhibited viral replication (Figures 4B–D). Similarly, the viral titres in the supernatant of cells transfected with siH2BE were decreased (Figure 4E). Compared with the control group, confocal microscopy revealed fewer PEDV virions in siH2BE-transfected cells (Figures 4F and G). Taken together, these data suggest that H2BE positively regulates PEDV replication.

### H2BE promotes PEDV replication but not by inhibiting the expression of interferons

**Figure 5:**
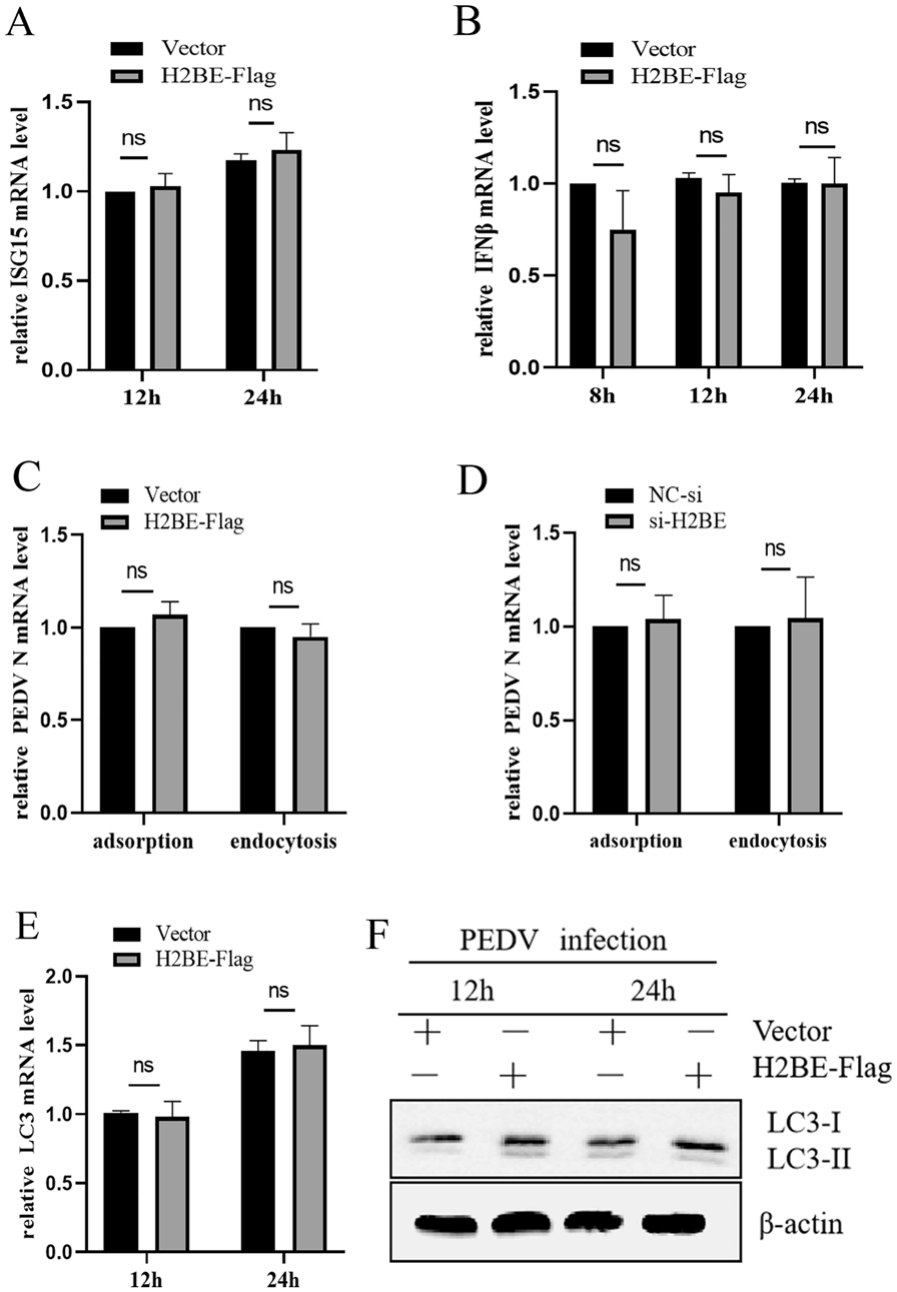
**H2BE has no effect on the expression of interferon.** Marc-145 cells were transfected with H2BE-Flag or an empty vector and incubated for 36 h, infected with PEDV at an MOI of 1.0 and harvested 12 h and 24 h later. **A** ISG15 mRNA was detected and analysed by qPCR. **B** IFN-β mRNA was detected and analysed by qPCR. Marc-145 cells were transfected with H2BE-Flag (si-H2BE) or an empty vector (NC-si), incubated for 36 h and then infected with PEDV at an MOI of 1.0. Then, the cells were placed in 4 ℃ adsorption for 1 h. Marc-145 cells were transfected with H2BE-Flag (si-H2BE) or an empty vector (NC-si), incubated for 36 h and then infected with PEDV at an MOI of 1.0. Then, the cells were placed at 4 ℃ for an hour and endocytosed at 37 ℃ for an hour. **C**, **D** PEDV N mRNA was measured by qPCR. (E) LC3II mRNA was detected and analysed by qPCR. **F** The expression levels of LC3II protein were measured by Western blotting. β-Actin was assessed as an internal control. The results are representative of three independent experiments. The data are presented as the mean ± SD, *n* = 3, (*P < 0.05; **P < 0.01).

The aforementioned experiments revealed that the overexpression of H2BE promotes the replication of PEDV. It had previously been reported that extrachromosomal cytoplasmic histone H2B was involved in aberrant self or non-self dsDNA recognition and induction of IFN-β [16]. To investigate whether H2BE promotes viral replication in Marc-145 cells by affecting type I interferon levels, we transfected H2BE overexpression plasmids into Marc-145 cells. The data showed that overexpression of H2BE did not affect the mRNA expression of ISG15 ubiquitin-like modifier (ISG15) (Figure 5A) or interferon β (IFN-β) (Figure 5B). In addition, we found that H2BE did not affect the adsorption or endocytosis of PEDV by Marc-145 cells (Figures 5C and D). We examined the mRNA and protein levels of LC3II in H2BE-Flag-transfected cells by qPCR and Western blotting and found that H2BE cells did not affect the expression of LC3II that had been induced to expression by PEDV (Figures 5E and F). These results suggest that the promotion of PEDV replication by histone H2BE functions independent of interferon expression, adsorption, endocytosis or autophagy.

### Overexpression of H2BE inhibits PEDV-induced ER stress

**Figure 6:**
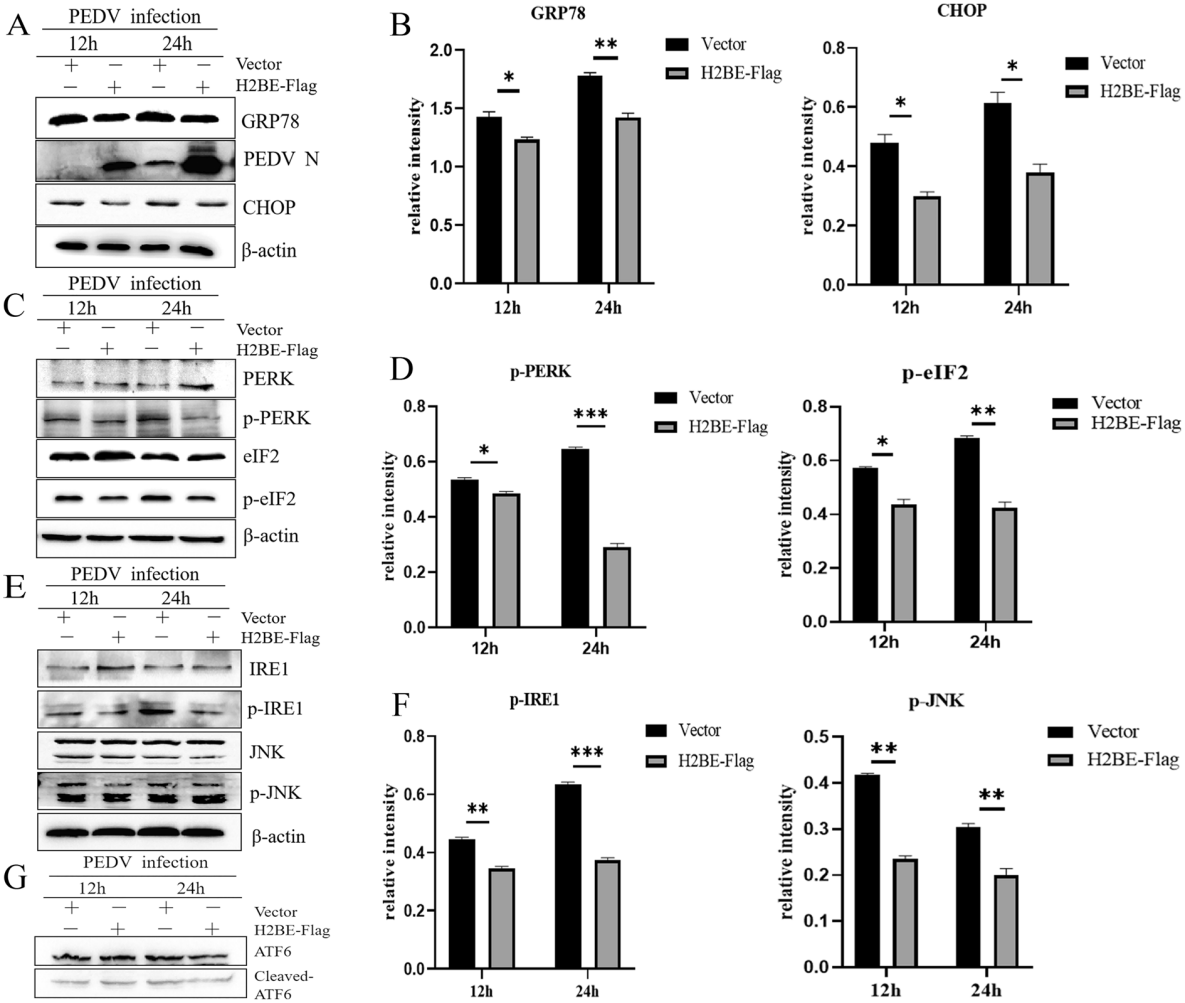
**Overexpression of H2BE alleviates PEDV-induced ER stress.** Marc-145 cells were transfected with H2BE-Flag or empty vector and then infected with PEDV at an MOI of 1.0 for 12 h and 24 h. **A** The protein expression levels of GRP78 and CHOP were measured by Western blotting. **B** The relative intensity represents GRP78 and CHOP protein levels normalized to β-actin. **C** The protein expression levels of PERK and eIF2 were measured by Western blotting. **D** The relative intensity represents phosphorylated PERK protein levels normalized to β-actin. The relative intensity represents phosphorylated eIF2 protein levels normalized to β-actin. **E** The protein expression levels of IRE1 and JNK were measured by Western blotting. **F** The relative intensity represents phosphorylated IRE1 protein levels normalized to that of β-actin. The relative intensity represents phosphorylated JNK protein levels normalized to that of β-actin. **G** The expression levels of ATF6 and cleaved ATF6 protein were measured by Western blotting. The results are representative of three independent experiments. The data are presented as the mean ± SD, *n* = 3, (*P < 0.05; **P < 0.01).

During viral infection, the synthesis and amount of viral proteins in the cell increase, increasing the viral burden in the ER and further increasing the accumulation of unfolded and misfolded proteins in the ER. The sudden increase in protein load in the ER induces ER stress. In response to ER stress, the unfolded protein response (UPR) is triggered to re-establish ER homeostasis [8]. Therefore, we wanted to determine whether H2BE affects PEDV replication in a manner related to ER stress. To test this possibility, we transfected Marc-145 cells with H2BE-Flag for 36 h, infected the cells with PEDV, and collected cells 12 h and 24 h later. Subsequently, Western blotting was performed to measure the expression of ER stress-related proteins. We found that H2BE-Flag-overexpressing cells showed decreased levels of the GRP78 protein (Figures 6A and B), phosphorylated PERK, phosphorylated eIF2 (Figures 6C and D), phosphorylated IRE1 and phosphorylated JNK (Figures 6E and F) 12 h and 24 h after infection, compared to levels in the empty vector group. Compared with the control group, no change in the abundance of the ATF6 protein was found in the H2BE overexpression group (Figure 6G). These results suggest that overexpression of H2BE suppressed PEDV-induced ER stress.

### Knockdown of H2BE exacerbates PEDV-induced ER stress

**Figure 7:**
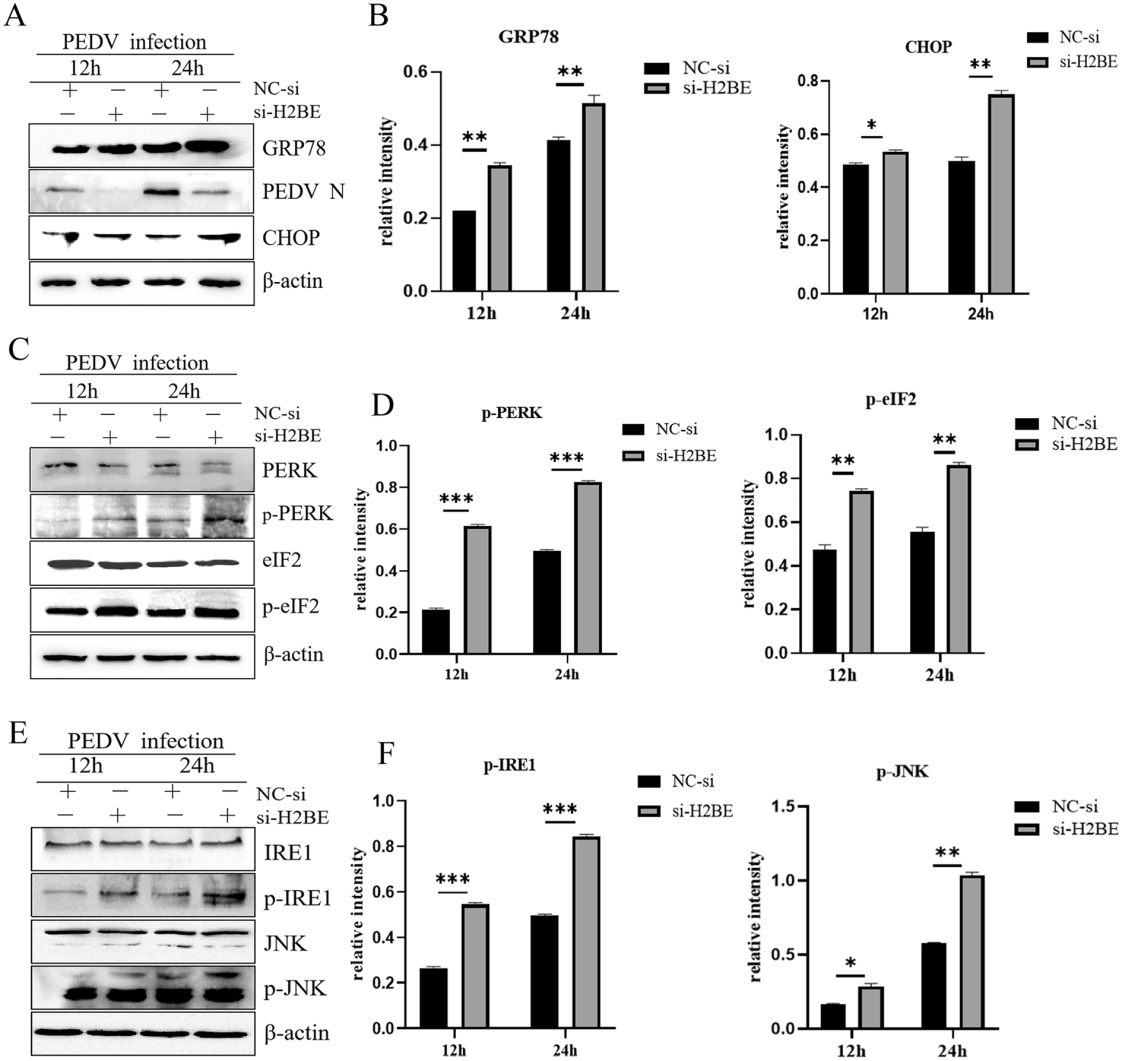
**Knockdown of H2BE exacerbates PEDV-induced ER stress.** Marc-145 cells were transfected with si-H2BE or NC-si and then infected with PEDV at an MOI of 1.0 and incubated for 12 h and 24 h. **A** The protein expression levels of GRP78 and CHOP were measured by Western blotting. **B** The relative intensity represents GRP78 and CHOP protein levels normalized to that of β-actin. **C** The protein expression levels of PERK and eIF2 were measured by Western blotting. **D** The relative intensity represents phosphorylated PERK protein levels normalized to the level of β-actin. The relative intensity represents phosphorylated eIF2 protein levels normalized to the level of β-actin. **E** The protein expression levels of IRE1 and JNK were measured by Western blotting. **F** The relative intensity represents phosphorylated IRE1 protein levels normalized to the level of β-actin. The relative intensity represents phosphorylated JNK protein levels normalized to the level of β-actin. The results are representative of three independent experiments. The data are presented as the mean ± SD, n = 3, (*P < 0.05; **P < 0.01).

To elucidate the role of H2BE in PEDV-induced ER stress, we examined changes in PEDV-induced ER stress in siH2BE-transfected MARC-145 cells. siH2BE was transfected into Marc-145 cells, which were cultured for 36 h and then infected with PEDV before a final culture for 12 h and 24 h. Protein levels of GRP78, PERK, eIF2, IRE1 and JNK were analysed by Western blotting. In contrast to the results of the overexpression experiment, the levels of the GRP78 protein (Figures 7A and B), phosphorylated PERK, phosphorylated eIF2 (Figures 7C and D), phosphorylated IRE1 and phosphorylated JNK (Figures 7E and F) in the siH2BE cells were significantly higher than those in the control group 12 h and 24 h after PEDV infection. These data suggest that knockdown of H2BE promotes PEDV-induced ER stress.

### H2BE inhibits PEDV-induced apoptosis

**Figure 8:**
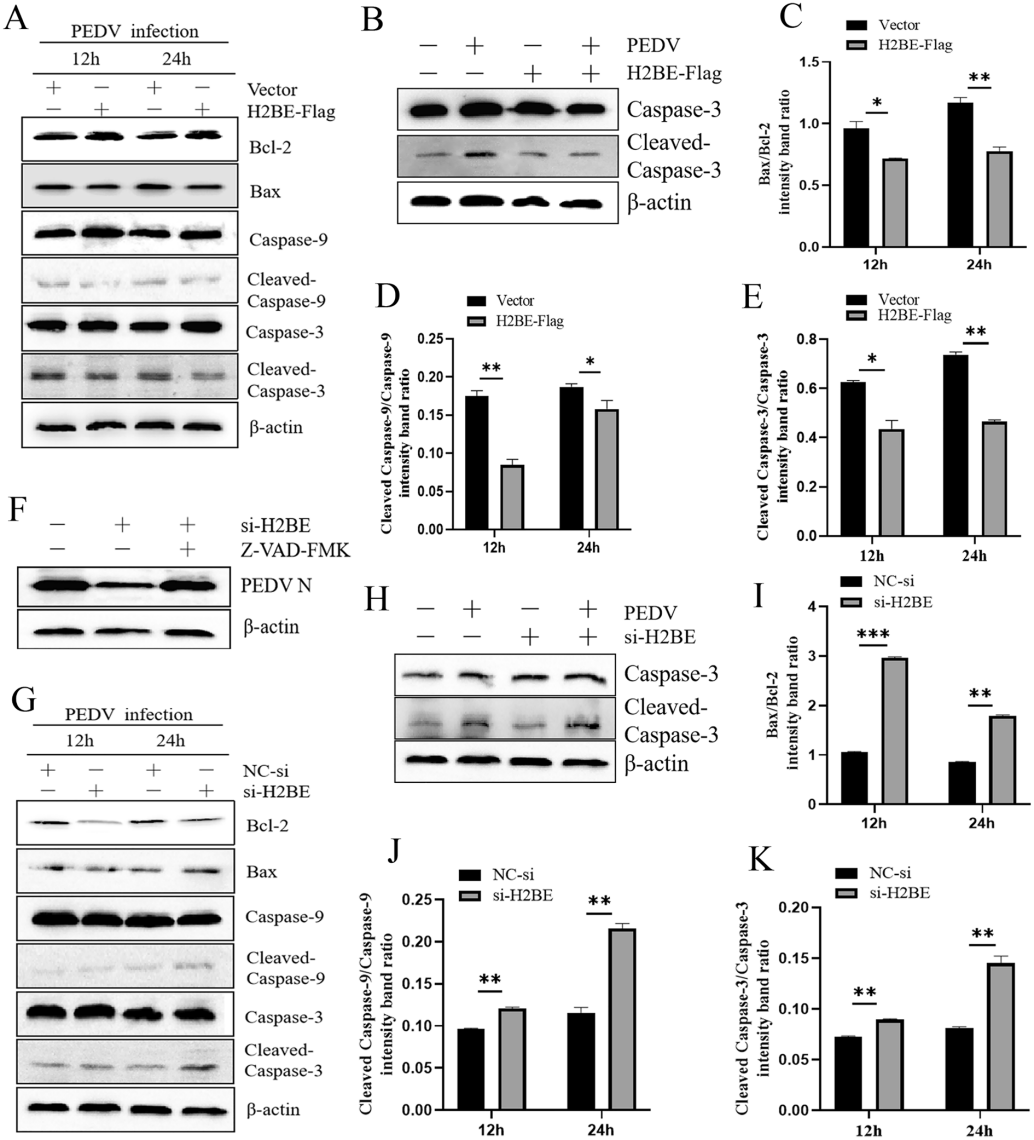
**H2BE inhibits PEDV-induced apoptosis**. Marc-145 cells were transfected with H2BE-Flag or an empty vector, infected with PEDV at an MOI of 1.0 and incubated for 12 h and 24 h. **A** The expression levels of Bcl-2, Bax, caspase-9, and caspase-3 were measured by Western blotting. Marc-145 cells were transfected with H2BE-Flag or empty vector for 36 h and infected with PEDV. Cells were harvested 24 h after PEDV infection. **B** The expression levels of caspase-3 and cleaved caspase-3 protein were measured by Western blotting. **C** The intensity represents Bax normalized to Bcl-2. **D** The intensity represents the level of cleaved caspase-9 normalized to that of caspase-9. **E** The intensity represents the level cleaved caspase-3 normalized to that of caspase-3. Marc-145 cells were transfected with H2BE-Flag or an empty vector, incubated for 36 h and pretreated with Z-VAD-FMK 1 h before PEDV infection. **F** The expression levels of PEDV N protein were measured by Western blotting. Marc-145 cells were transfected with si-H2BE or NC-si, infected with PEDV at an MOI of 1.0 and then incubated for 12 h and 24 h. **G** The expression levels of Bcl-2, Bax, caspase-9 and caspase-3 were measured by Western blotting. Marc-145 cells were transfected with si-H2BE or NC-si, incubated for 36 h, and infected with PEDV. Cells were harvested 24 h after PEDV infection. **H** The expression levels of caspase-3 and cleaved caspase-3 protein were measured by Western blotting. **I** The intensity represents Bax normalized to Bcl-2. **J** The intensity represents the level of cleaved caspase-9 normalized to that of caspase-9. **K** The intensity represents the level of cleaved caspase-3 normalized to that of caspase-3. The results are representative of three independent experiments. The data are presented as the mean ± SD, *n* = 3, (*P < 0.05; **P < 0.01).

If long-term ERS/UPR signalling cannot effectively relieve ER stress, then the UPR is activated to maintain the protein balance of the whole cell, and the expression of C/EBP homologous protein (CHOP) is activated to induce cells to undergo apoptosis [17]. From the aforementioned results, overexpression of H2BE inhibits PEDV-induced ER stress and downregulates CHOP expression (Figure 6A). We next investigated the effect of H2BE on cell apoptosis. We detected and analysed the expression levels of some apoptosis-related proteins by Western blotting. As we had suspected, the data showed that the ratios of Bax/Bcl-2, cleaved caspase-9 and cleaved caspase-3 were significantly reduced in cells transfected with H2BE-Flag (Figures 8A, C–E). We observed the opposite outcomes in cells transfected with siH2BE (Figures 8G, I–K). These results suggest that overexpression of H2BE inhibited PEDV-induced apoptosis, whereas knockdown of H2BE increased the rate of PEDV-induced apoptosis. In addition, we investigated whether H2BE induces apoptosis. Analysis of cleaved caspase-3 protein levels by Western blotting showed that overexpression or knockdown of H2BE did not affect the apoptosis rate of the Marc-145 cells (Figures 8B and H). H2BE cells inhibited apoptosis only when stimulated by virus. To confirm this relationship, the caspase inhibitor Z-VAD-FMK was used to inhibit apoptosis in siH2BE-transfected cells. si-H2BE showed a weakened inhibitory effect on PEDV (Figure 8F). Therefore, the results indicated that H2BE affected PEDV replication by regulating apoptosis. From these results, we conclude that H2BE promoted PEDV replication in Marc-145 cells by inhibiting apoptosis.

### H2BE effectively inhibits ER stress-mediated apoptosis

**Figure 9:**
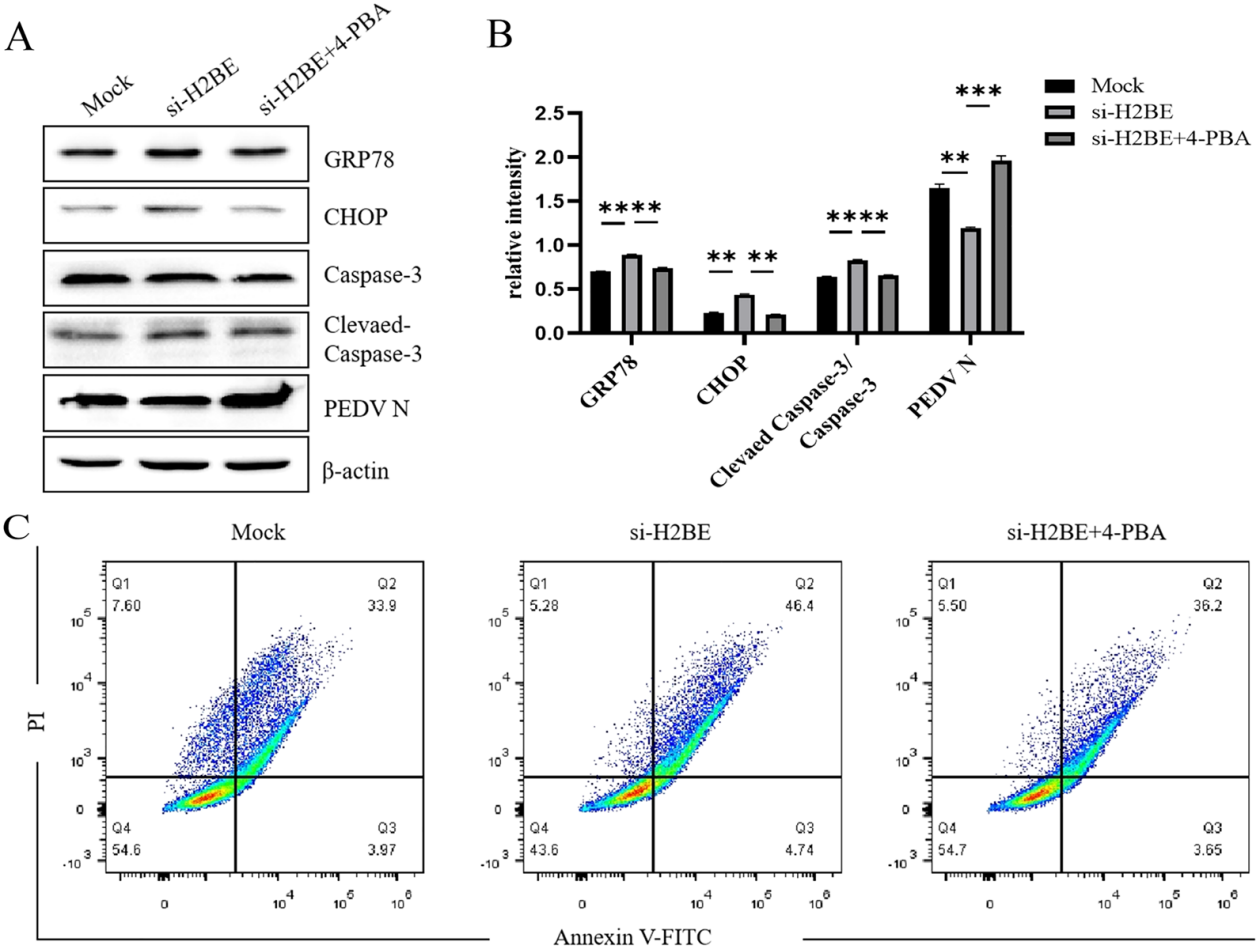
**H2BE effectively inhibits apoptosis in the ER stress pathway**. Marc-145 cells were transfected with si-H2BE or NC-si, incubated for 36 h, pretreated with the ER stress inhibitor 4-PBA for 1 h, infected with PEDV at an MOI of 1.0, and incubated for 24 h. **A** The expression levels of GRP78, CHOP, cleaved caspase-3 and PEDV N protein were measured by Western blotting. **B** The intensity represents GPR78, CHOP and PEDV N protein levels normalized to the level of β-actin, and the intensity represents the level of cleaved caspase-3 normalized to that of caspase-3. **C** Marc-145 cells were transfected with si-H2BE or NC-si, infected with PEDV at an MOI of 1.0 and incubated for 24 h, and stained with Annexin V and PI. The results are representative of three independent experiments. The data are presented as the mean ± SD, *n* = 3, (*P < 0.05; **P < 0.01).

Knockdown of H2BE can upregulate CHOP expression, promote apoptosis and inhibit viral replication. To validate the mechanism by which H2BE promotes viral replication through the inhibition of ER stress-mediated apoptosis, siH2BE was transfected into Marc-145 cells for 36 h, and then, the ER stress inhibitor 4-PBA was used to pretreat Marc-145 cells for 1 h before infection with PEDV. The treated and infected cells were harvested 24 h post-infection. The expression levels of related proteins were analysed by Western blotting. As shown in Figures 9A and B, the protein levels of GRP78, CHOP and cleaved caspase-3 were increased in H2BE-knockdown cells, while PEDV N protein levels were decreased, and the addition of the ER stress inhibitor 4-PBA restored the levels of these proteins. A flow cytometry assays showed that the addition of the ER stress inhibitor 4-PBA inhibited the promotion of PEDV-induced apoptosis that had been induced by siH2BE (Figure 9C). These results suggested that knockdown of H2BE effectively promotes ER stress-mediated apoptosis, thereby inhibiting PEDV replication, and that H2BE exerts positive regulation of PEDV through the inhibition of ER stress-mediated apoptosis.

### Amino acids 1–28 of H2BE are essential for promoting PEDV replication

**Figure 10:**
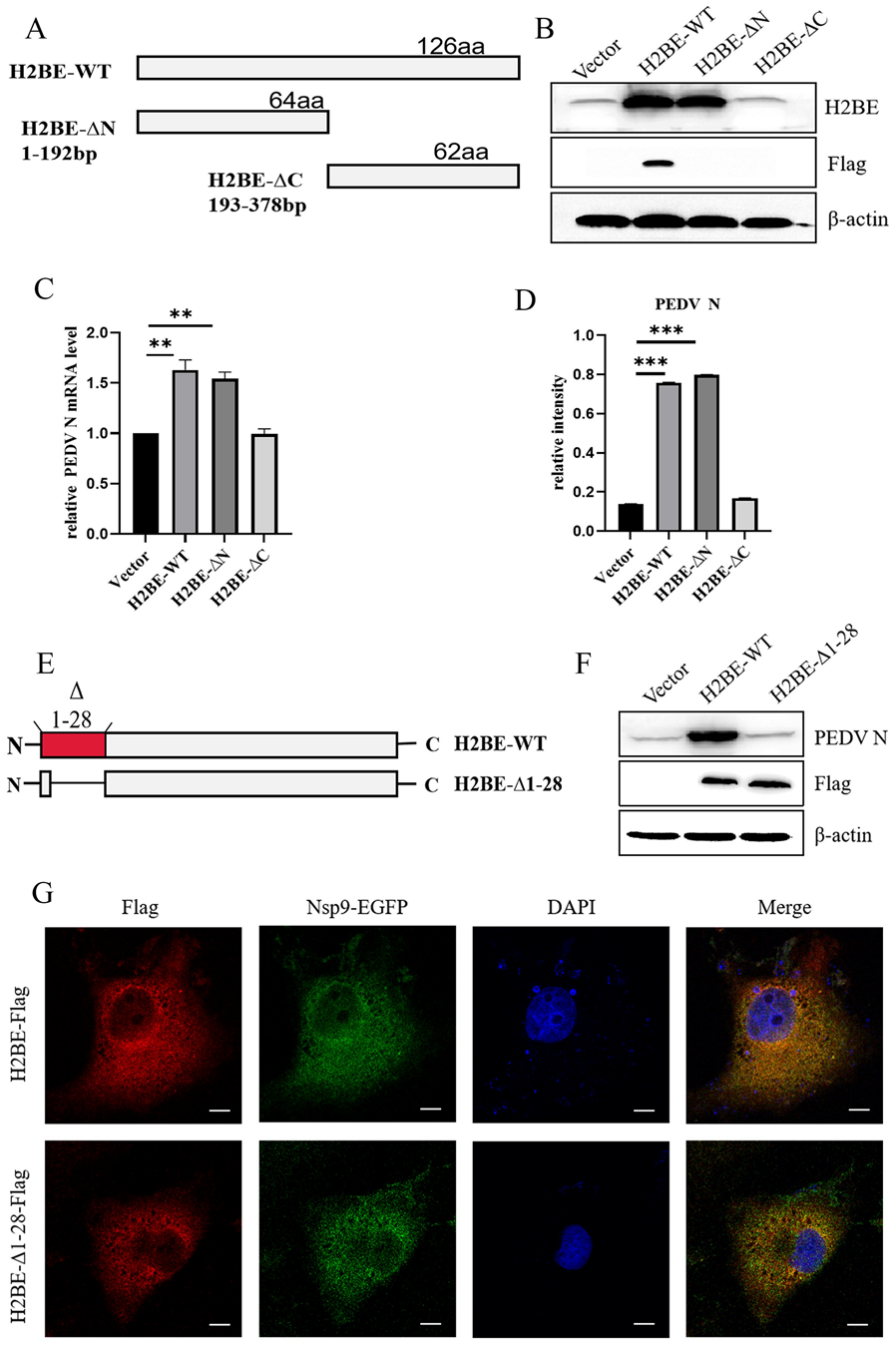
**Nuclear shuttle function of H2BE is essential for PEDV replication.** **A** Full-length H2BE, C-terminal deletion mutant H2BE-ΔN and N-terminal deletion mutant H2BE-ΔC. Marc-145 cells were separately transfected with an empty vector, H2BE-Flag, H2BE-ΔN-Flag, and H2BE-ΔC-Flag and then infected with PEDV at an MOI of 1.0 and incubated for 12 h. **B** The expression levels of PEDV N protein were measured by Western blotting. **C** PEDV N mRNA was measured by q-PCR. β-Actin was used as the internal control. **D** The intensity represents PEDV N protein levels normalized to the level of β-actin. The results are representative of three independent experiments. The data are presented as the mean ± SD, *n* = 3, (* *P* < 0.05; ** *P* < 0.01). **E** H2BE deletion mutants with amino acids 1–28 deleted. **F** The expression levels of PEDV N protein were analysed by Western blotting. H2BE-Flag and H2BE-Δ1-28-Flag were cotransfected with Nsp9-EGFP into Marc-145 cells, which were stained with DAPI (blue) and antibodies against Flag (red). **G** The subcellular localization of H2BE-WT and H2BE-Δ1-28 with Nsp9-EGFP.

To define the specific structural region where histone H2BE promotes PEDV replication, we constructed the N-terminal H2BE deletion mutant H2BE-ΔC-Flag and the C-terminal H2BE deletion mutant H2BE-ΔN-Flag (Figure 10A) and found that H2BE-ΔC-Flag played no role in promoting PEDV replication after transfection into Marc-145 cells (Figures 10B–D). This result indicates that the functional domain of H2BE that promotes PEDV replication is located mainly in the N-terminus. Studies have shown that the N-terminal 1–28 amino acid sequence in H2BE constitutes a region on the protein surface that is unaffected by changes to the DNA sequence [13]. We constructed the amino acid 1–28 deletion mutant plasmid H2BE-Δ1-28-Flag (Figure 10E). Next, H2BE-Flag and H2BE-Δ1-28-Flag were transfected into Marc-145 cells, and we found that when amino acids 1–28 were deleted, the promoting effect of H2BE on PEDV replication disappeared, in contrast to the effect of wild-type H2BE (Figure 10F). The subcellular colocalization of H2BE-Δ1-28-Flag, H2BE-Flag and Nsp9-EGFP was examined by indirect immunofluorescence assay. We found that H2BE-Δ-1-28-Flag colocalized with Nsp9-EGFP (Figure 10G). The results showed that the N-terminal 1–28 amino acid sequence in H2BE is required for the promotion of virus replication but does not affect the interaction of H2BE with Nsp9. These results indicate that the H2BE protein relies on the N-terminal 1–28 amino acid sequence to promote PEDV replication.

## Discussion

**Figure 11:**
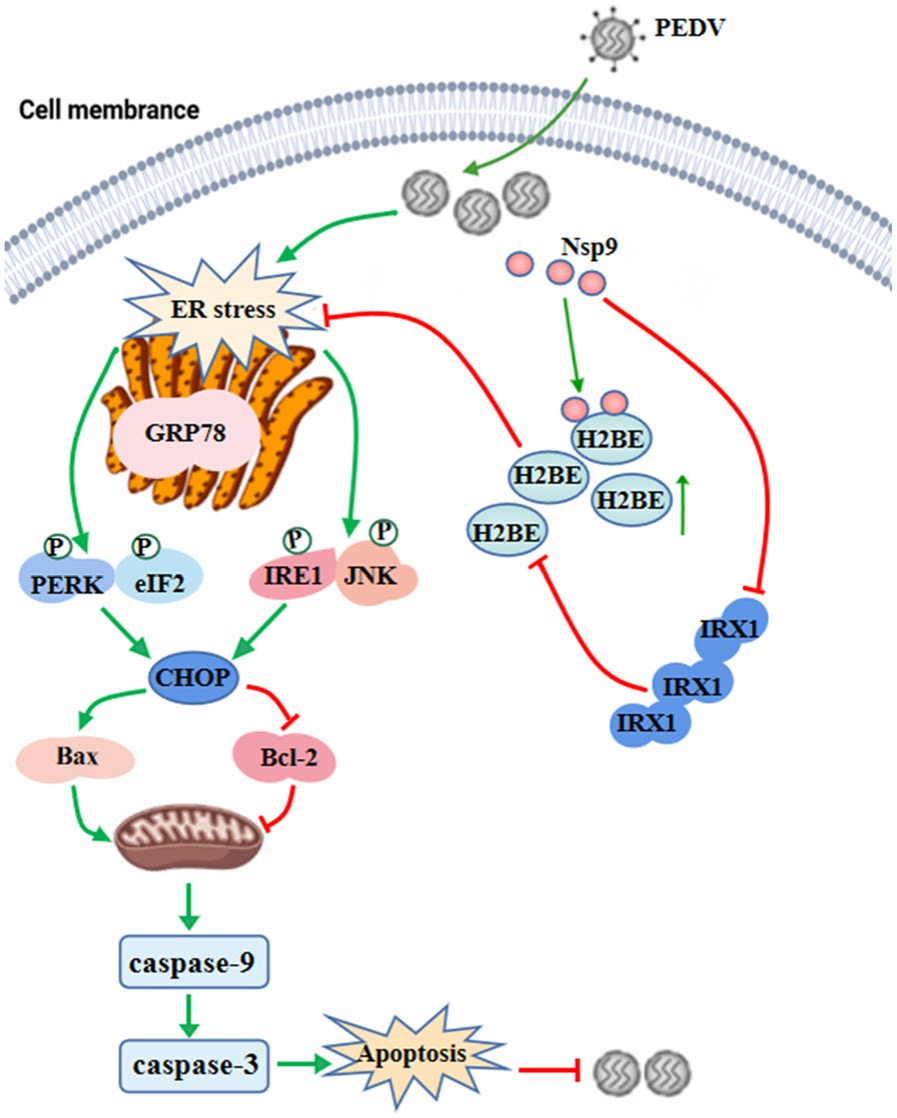
**PEDV upregulates H2BE expression to inhibit ER stress-mediated apoptosis**. PEDV Nsp9 upregulates H2BE expression by regulating IRX1 expression. Overexpression of H2BE inhibits PEDV-induced activation of the ER stress-driven PERK and IRE1 pathways and suppresses CHOP expression and apoptosis. Inhibition of apoptosis promotes PEDV replication.

Understanding the host response to PEDV is crucial for generating new preventive or therapeutic approaches for this destructive disease [18]. Some PEDV proteins have been shown to contribute to their cell proliferation by interacting with host signalling molecules [19, 20]. In this study, we constructed a PEDV Nsp9 expression plasmid that was transferred into Marc-145 cells. H2BE was predicted to interact with PEDV Nsp9, and this hypothesis was tested using co-IP and mass spectrometry. Immunoprecipitation and confocal microscopy were then performed to demonstrate that Nsp9 interacts with H2BE cells (Figure 11).

Changes in H2BE expression were observed in some virus-infected cells, such as latent human herpes simplex virus type-1 (HSV-1) infection, which upregulates H2BE expression mediated through the downregulation of ICP0 protein levels [21]. In the current study, we found that H2BE expression was upregulated in PEDV Nsp9-overexpressing Marc-145 cells. Our hypothesis suggested that PEDV interacts with some host factors to upregulate the expression of H2BE, promoting viral infection. The Iroquois homeobox protein 1 (IRX1) tumour suppressor gene is a cancer susceptibility locus. H2BE is identified as a direct IRX1 target gene [13]. H2BE expression can be regulated by inhibiting IRX1. In addition, downregulation of IRX1 has been shown to upregulate the expression of H2BE [14]. In this study, Western blotting and qPCR revealed that PEDV Nsp9 upregulates H2BE expression by suppressing the expression of IRX1. Overexpression of IRX1 restores H2BE levels.

Previous studies have shown that H2BE plays an important role in many diseases, including innate immunity regulation and apoptosis regulation. For example, downregulation of H2BE expression promotes neuronal cell death [22]. Upregulation of H2BE in IDC tissues inhibits apoptosis and facilitates tumour growth [14]. Knockdown of H2B inhibits IFN-β production in KSHV-, HSV-1- and EBV-infected cells [15]. However, the relationship between H2BE and PEDV replication has not been reported. In this study, we observed that overexpression of H2BE enhanced PEDV replication and that knockdown of H2BE inhibited PEDV replication. In vitro, IFN-β and ISG15 directly confers resistance to viral infections. Our results showed that the mRNA levels of IFN-β and ISG15 were not affected by the overexpression of H2BE. In experiments with the overexpression or knockdown of H2BE, H2BE was not involved in viral adsorption or viral endocytosis by cells. These results suggest that H2BE promotes PEDV replication through pathways not dependent on absorption or endocytosis.

Viruses need to produce a large number of structural and nonstructural proteins in the ER to complete their life cycle. Previous studies have shown that the structural proteins E and N of PEDV and the nonstructural protein ORF3 can trigger ER stress [23–26]. When ER stress is severe, prolonged or not properly resolved, the UPR eventually induces the expression of CHOP, inducing apoptosis [27, 28]. HCV activates the UPR and induces apoptosis by upregulating the expression of GADD153/CHOP [29]. JEV induces the UPR and initiates apoptosis by inducing CHOP [30]. Several studies have shown that the PERK and IRE1 pathways remain the main pathways for the induction of CHOP expression, which is also governed by the transition between the unphosphorylated and phosphorylated forms of eIF2 during the UPR [31]. In response to ER stress, high expression of CHOP promotes caspase activation through the upregulation of the proapoptotic protein Bax and downregulation of the antiapoptotic protein Bcl-2, leading to apoptosis. Our previous studies have shown that PEDV infection induces the initiation of intrinsic apoptosis pathways [12, 32]. Our present study indicates that overexpression of H2BE promotes PEDV replication, downregulates CHOP expression in the PERK, eIF2, IRE1, and JNK pathways, decreases the expression of Bax, cleaved caspase-9 and cleaved caspase-3 and increases the expression of Bcl-2. Moreover, knockdown of H2BE increases the expression of p-PERK, p-eIF2, p-IRE1, p-JNK, CHOP, Bax, cleaved caspase-9 and cleaved caspase-3 and decreases the expression of Bcl-2. Furthermore, we found that knockdown of H2BE promotes the expression of GRP78 and CHOP and increases activation and that the addition of the ER stress inhibitor 4-PBA inhibits these activities. These findings suggest that H2BE promotes PEDV replication through the inhibition of ER stress-mediated apoptosis.

Studies have shown that DNASE1L3 damages angiogenesis by interacting with H2BE, and the loss of H2BE amino acids 1–28 results in the of H2B2 interaction with DNASE1L3 [13]. In our study, we constructed H2BE-Δ1-28-Flag, a vector carrying a mutation of H2BE with amino acids 1–28 deleted. H2BE-Δ1-28-Flag was transfected into Marc-145 cells, and deletion of H2BE amino acids 1–28 failed to promote the expression of PEDV. These results indicate that amino acids 1–28 may be the key residues that induce H2BE-dependant PEDV replication.

In summary, our study demonstrates for the first time that PEDV Nsp9 interacts with host protein H2BE. We provide evidence that Nsp9 upregulates the expression of H2BE. H2BE promotes PEDV replication by inhibiting ER stress-mediated apoptosis. These results provide new insights into the molecular mechanisms of PEDV–host interactions, which might indicate new therapeutic targets for the control of PEDV infection.

## Supplementary Information

**Additional file 1. The intensity analysis of the Flag protein in Figure ****3****E.** The intensity of Flag protein increased with an increase in the transfection dose of the H2BE overexpression plasmid.

## Data Availability

The data analysed during the current study are available from the corresponding author upon reasonable request.

## Abbreviations

TCID_50_: 50% Tissue culture infective dose
CHOP: C/Ebp-homologous protein
