# Supplementary figures and images for: The novel Nsp9-interacting host factor H2BE promotes PEDV replication by inhibiting endoplasmic reticulum stress-mediated apoptosis
Source: Vet Res. 2023 Mar 22;54:27. doi: 10.1186/s13567-023-01158-w (PMC10035214; doi:10.1186/s13567-023-01158-w)

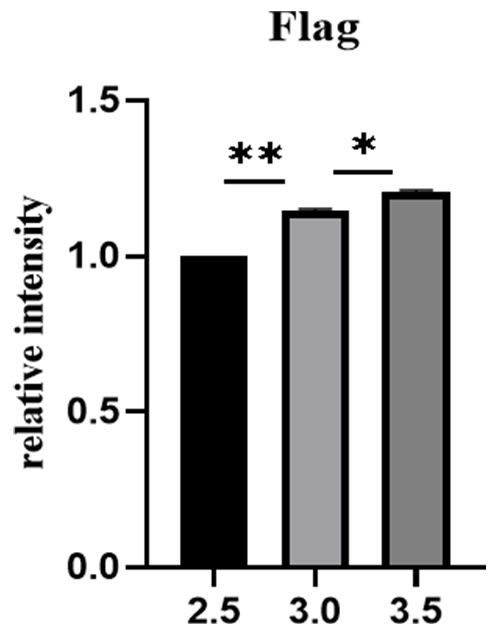

Supplement: Supplementary file 1 — Additional file 1. The intensity analysis of the Flag protein in Figure 3E. The intensity of Flag protein increased with an increase in the transfection dose of the H2BE overexpression plasmid. [file 13567_2023_1158_MOESM1_ESM.tif]
